# Reactive Astrocyte‐Associated IL‐33 Signaling Contributes to a Protective Neuroimmune State During Chronic *Toxoplasma gondii* – *Plasmodium berghei* Coinfection

**DOI:** 10.1002/glia.70257

**Published:** 2026-11-09

**Authors:** Inès Leleu, Tarun Keswani, Fabien Herbert, Capucine Picavet, Manon Arnaud, Sabrina Marion, Corine Glineur, Sylviane Pied

**Affiliations:** ^1^ Univ. Lille, CNRS UMR 9017, Inserm U1019, Center for Infection and Immunity of Lille (CIIL), Institut Pasteur de Lille Lille France; ^2^ Center for Immunology and Inflammatory Diseases Massachusetts General Hospital Boston Massachusetts USA; ^3^ Institute of Functional Genomics (IGF), University of Montpellier, CNRS UMR 5203, Inserm U1191 Montpellier France

**Keywords:** astrocytes, cerebral malaria, coinfection, IL‐33, neuroinflammation

## Abstract

Cerebral malaria (CM) is a lethal neuroinflammatory syndrome characterized by blood–brain barrier disruption and excessive immune‐mediated neuronal damage. Astrocytes are emerging as key regulators of CNS homeostasis, yet their contribution to disease tolerance during polymicrobial infections remains poorly understood. Here, we show that chronic *Toxoplasma gondii* (*Tg*) infection protects against experimental cerebral malaria (ECM) induced by *Plasmodium berghei* ANKA (*Pb*A) by establishing a regulated neuroimmune environment rather than reducing cerebral parasite burden. This protective phenotype required chronic cerebral *T. gondii* infection and was associated with a distinct neuroinflammatory transcriptional profile characterized by enhanced TGF‐β and IL‐33/ST2 expression. At the cellular level, chronic *Tg* infection remodeled the astrocyte compartment, generating reactive astrocytes displaying an intermediate inflammatory phenotype characterized by the co‐expression of inflammatory (CXCL‐10, MHC‐I) and regulatory (TGF‐β) markers. Reactive astrocytes represented one of the major IL‐33‐expressing cell populations in the brains of *Tg*‐infected and coinfected mice and were associated with recruitment of ST2‐expressing group 2 innate lymphoid cells (ILC2s), which expressed IL‐5 and IL‐13 in the brain. In IL‐33‐deficient mice, cerebral ILC2 recruitment was markedly reduced and protection against ECM was only partially maintained, indicating that the IL‐33/ST2 axis contributes to, but is not solely responsible for, disease tolerance during coinfection. Our findings identify reactive astrocytes as key components of a regulated neuroimmune environment associated with protection against ECM during chronic *T. gondii* coinfection. These results highlight astrocyte‐associated IL‐33/ST2 signaling and ILC2 recruitment as components of a multicellular neuroimmune network that contribute to disease tolerance during polymicrobial CNS infections.

## Graphical abstract

Chronic *Toxoplasma gondii* infection establishes a regulated neuroimmune environment that protects against experimental cerebral malaria without reducing cerebral parasite burden. Coinfection is associated with the emergence of reactive astrocytes displaying an intermediate inflammatory phenotype characterized by the co‐expression of CXCL‐10 and TGF‐β, together with preserved blood–brain barrier integrity and reduced neuroinflammation. Reactive astrocytes represent one of the major IL‐33‐expressing cell populations and are associated with increased recruitment of ST2^+^ ILC2s, contributing to a multicellular neuroimmune network linked to disease tolerance. Graphical abstract created using BioRender.com.

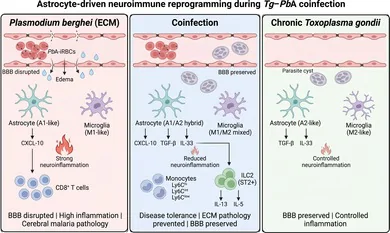

## 1 Introduction

Coinfections involving neurotropic parasites are frequent in endemic regions and represent an underexplored determinant of CNS disease outcome (Galani et al. 2021; Hussein et al. 2022; Luz et al. 2018). Once considered immune‐privileged, the brain is now recognized as a highly dynamic immunological environment in which glial cells actively shape inflammatory responses. Neuroparasitic infections such as cerebral malaria (CM), caused by *Plasmodium falciparum*, and toxoplasmosis, caused by *Toxoplasma gondii* (*Tg*), are major contributors to neurological morbidity worldwide, often leading to long‐term cognitive impairment or death (Hellani et al. 2024; WHO 2023; Alloo et al. 2022). Epidemiological overlap between these pathogens suggests that coinfections are likely common, yet their impact on CNS immunity remains poorly understood (WHO 2023; Achonduh‐Atijegbe et al. 2016; Kamuyu et al. 2014).

CM, the most severe complication of *Plasmodium falciparum* infection, is characterized by acute encephalopathy with coma and seizures (WHO 2023). The pathogenesis of CM involves both sequestration of infected red blood cells (iRBCs) within cerebral microvessels and a dysregulated neuroinflammatory response, accompanied by BBB disruption (Baptista et al. 2010). In experimental CM (ECM) induced by *Plasmodium berghei ANKA* (*Pb*A) in C57BL/6 mice, disease progression is driven by excessive production of pro‐inflammatory mediators including CXCL‐10 (C‐X‐C motif chemokine ligand‐10), CCL‐2 (C‐C motif chemokine ligand‐2), TNF‐α (tumor necrosis factor), and interferon gamma (IFN‐γ), leading to recruitment of pathogenic αβCD8^+^ T cells into the brain (Dalko et al. 2016; Shrivastava et al. 2017; Keswani et al. 2020; Leleu et al. 2022; Belnoue et al. 2002; Bagot et al. 2004; Boubou et al. 1999). These events culminate in vascular damage, BBB breakdown, and neurological failure (Bagot et al. 2004; Haque et al. 2011; Potter et al. 2006).

Astrocytes are increasingly recognized as key regulators of this process (Bugiani et al. 2022). Upon *Plasmodium* infection, they undergo profound transcriptional and functional remodeling, contributing to both chemokine production and modulation of immune cell recruitment (Dalko et al. 2016; Shrivastava et al. 2017; Fan and Huo 2021). Recent studies demonstrated that *PbA*‐derived microvesicles can reprogram astrocytes through a *Rubicon‐dependent non‐canonical autophagy pathway*, leading to *astrocyte senescence and enhanced production of chemokines such as CXCL‐10* (Hellani et al. 2024; Leleu et al. 2022). Importantly, astrocytes exhibit a broad spectrum of activation states and function as central regulators of neuroimmune responses, dynamically balancing neurotoxicity and neuroprotection within the CNS (Fan and Huo 2021).

In contrast, chronic *Tg* infection induces a more regulated neuroimmune environment (Sanchez and Besteiro 2021; Yang et al. 2024; Ross et al. 2022). During persistent infection, astrocytes limit parasite dissemination while simultaneously promoting tissue protection through the production of CXCL‐10, CCL‐2, and interleukin‐6 (IL‐6) and TGF‐β (Blanchard et al. 2015; Wilson and Hunter 2004; Strack et al. 2002; Halonen 2009; Nasuhidehnavi and Yap 2021). In this context, astrocytes adopt a predominantly protective phenotype that promotes immune containment while preserving CNS structural and functional integrity (Cekanaviciute et al. 2014). Importantly, *Tg* infection also induces astrocytic production of IL‐33, a nuclear alarmin released upon cellular stress. IL‐33 signals through its receptor ST2 (Suppression of Tumorigenicity 2) to coordinate the recruitment of protective immune populations, including the group 2 innate lymphoid cells (ILC2s), IFN‐γ–producing T cells, and myeloid cells (Still et al. 2020; Clark et al. 2021). Rather than acting as a simple pro‐inflammatory cytokine, IL‐33 functions as a central organizer of local neuroimmune circuits that coordinate communication between astrocytes and recruited immune cells.

Given the shared CNS tropism of *Plasmodium* and *Toxoplasma*, their coexistence raises the possibility that pre‐existing neuroinfections may reshape susceptibility to severe malaria. Previous studies have suggested that chronic *Tg* infection can attenuate ECM severity, but the cellular and molecular mechanisms underlying this phenomenon remain unclear (Settles et al. 2014). In particular, the role of astrocytes as integrators of coinfection‐derived signals has not been directly investigated.

These observations suggest that astrocytes may act as integrative sensors of sequential infections, capable of translating prior immune experience into altered neuroinflammatory responses. Here, we hypothesized that chronic *Toxoplasma gondii* infection establishes a regulated neuroimmune environment that promotes disease tolerance and protects against ECM without necessarily reducing parasite burden. We further hypothesized that this protective state is associated with astrocyte remodeling, leading to the emergence of reactive astrocytes displaying an intermediate inflammatory phenotype together with enhanced IL‐33/ST2 expression. Using a murine model of *Tg*‐*Pb*A coinfection, we investigate how chronic cerebral *T. gondii* infection reshapes astrocytes' responses and the associated neuroimmune landscape underlying protection against ECM.

## 2 Materials and Methods

### 2.1 Mice

Female age‐matched C57BL/6JRj (6–8 weeks, Janvier Laboratories, France) and IL‐33 knockout (IL‐33^−/−^) mice (Duez et al. 2020) were housed under specific pathogen‐free conditions at the Institut Pasteur de Lille. All experiments were conducted in accordance with institutional, national (Decree 87‐848), European (Directive 86/609/CEE), and NIH (A5476‐01) guidelines for animal care and were approved by the Ethical Committee of Institute Pasteur of Lille and the French Committee for Animal Health Care “Ministère de l'Agriculture et de la Pêche” (#22021‐2019091715283871).

### 2.2 *Toxoplasma gondii* Infection

Type II *T. gondii* tachyzoites (Prugniaud Pru‐Luc‐GFP strain) were maintained in human foreskin fibroblasts cultured in Dulbecco's Modified Eagle Medium (DMEM; Gibco; 11.885‐084) supplemented with 10% fetal bovine serum (FBS; Sigma‐Aldrich; F0926), glutamine (Gibco; 25030081), and penicillin/streptomycin (P/S; Gibco; 10378016). Mice were infected intraperitoneally with 1 × 10^3^ tachyzoites.

### 2.3 *T. gondii* Parasite Burden and Cyst Quantification

Genomic DNA (gDNA) was extracted from brain homogenate using the NucleoSpin Tissue DNA extraction kit (Macherey‐Nagel; 740952.5) according to the manufacturer's recommendations. gDNA was quantified using a Nanodrop and the number of parasites per μg of brain DNA was calculated by real‐time qPCR. Briefly, the *T. gondii* B1 gene was amplified using SYBR GreenER SuperMix for iCycler (Invitrogen; 11761500) with an MgCl_2_ concentration adjusted to 3.5 mM in a 30 μL reaction volume, 1 μg of total template DNA from each organ, 1.50 μL of 0.5 μM primer (sequences referencing in Table S1 and water for a final volume of 30 μL). Gene amplification was performed with an iCycler reverse transcription (RT)‐PCR (*Polymerase Chain Reaction*) machine (Bio‐Rad Laboratories) using a 10 min initial denaturation at 95°C, followed by 50 cycles that consisted of 15 s of denaturation at 95°C, 30 s of annealing at 60°C, and 30 s of extension at 72°C. A standard curve was generated using a known number of corresponding parasites. Cysts were enumerated from brain homogenates labeled with FITC (Fluorescein Isothiocyanate)‐conjugated *Dolichos biflorus* agglutinin (Vector Laboratories; FL‐1031) and visualized by fluorescence microscopy with a 20× objective.

### 2.4 Experimental Cerebral Malaria (ECM) Model

ECM was induced by intraperitoneal infection with 1 × 10^6^ *Pb*A‐iRBCs (ECM^+^; *Pb*A group in Figure 1A) (Keswani et al. 2020). Parasitaemia was assessed on Giemsa‐stained blood smears at 6.5 days post‐infection, when ECM symptoms typically developed.

**FIGURE 1:**
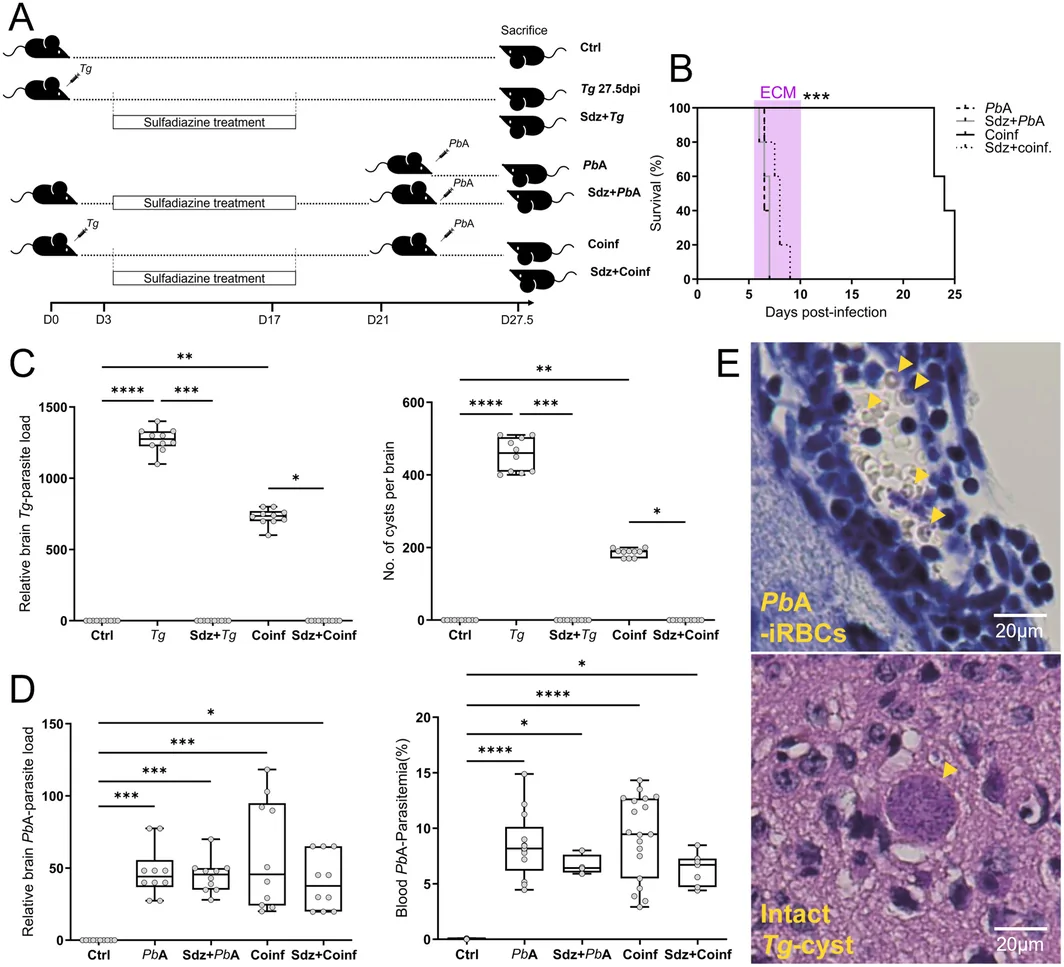
Chronic cerebral *Tg* infection is required for protection against ECM independently of *PbA* parasite burden. (A) Experimental design. Six‐week‐old C57BL/6 mice were infected intraperitoneally with 10^3^ *Tg* type II Pru‐GFP tachyzoites (day 0). Twenty‐one days later, corresponding to the establishment of chronic cerebral toxoplasmosis with brain tissue cysts, mice were challenged intravenously with 10^6^ *Pb*A‐iRBCs. Mono‐infected *Tg*, mono‐infected *Pb*A and non‐infected mice were included as controls. Sulfadiazine (SDZ; 1 g/L in drinking water) was administered from day 3 to day 17 after *Tg* infection to eliminate tachyzoites before brain colonization. An additional *Pb*A‐infected group also received SDZ treatment to assess the potential effect of the drug on ECM development. (B) Kaplan–Meier survival curves of *Pb*A‐infected and *Tg*–*Pb*A coinfected mice treated or not with SDZ. The shaded area indicates the ECM window (6–10 days post‐*Pb*A infection highlighted in violet). Survival curves were compared using the Mantel–Cox (log‐rank) test. (C) Assessment of chronic cerebral *Tg* infection. Left, relative cerebral *Tg* burden determined by RT‐qPCR analysis of the bradyzoite‐specific marker BAG‐1. Right, quantification of brain tissue cysts following FITC labeling of the cyst wall antigen on brain sections. No BAG‐1 signal or tissue cysts were detected in SDZ‐treated mice, confirming that sulfadiazine prevented the establishment of chronic cerebral *Tg* infection. (D) Assessment of *PbA* parasite burden. Left, relative cerebral *Pb*A burden quantified by RT‐qPCR targeting the *Pb18S* transcript. Right, peripheral parasitemia determined by microscopic examination of Giemsa‐stained thin blood smears during the ECM phase (6.5 days post‐*Pb*A infection). (E) Representative histological images of brain sections from *Tg*–*Pb*A coinfected mice stained with hematoxylin and eosin (H&E), showing *PbA*‐iRBCs sequestered within cerebral microvessels and *Tg* tissue cysts (yellow arrows) within the brain parenchyma. Scale bar = 20 μm. Data are presented as box‐and‐whisker plots showing the median (center line), the 25th–75th percentiles (box), and the minimum and maximum values (whiskers), with all individual data points displayed. Results are pooled from three independent experiments (*n* = 4–20 mice per experimental group). Statistical analyses were performed using the Kruskal–Wallis test followed by Dunn's multiple‐comparison test. Survival curves were compared using the Mantel–Cox (log‐rank) test. *p < 0.05; **p < 0.01; ***p < 0.001; ****p < 0.0001.

### 2.5 Coinfection Model

Five groups of mice were studied: (i) uninfected controls, (ii) *Pb*A‐only, (iii) *Tg*‐only, (iv) *Tg*–*Pb*A coinfected mice (infected with *Tg* for 21 days before *Pb*A challenge, as described by Settles et al. 2014), and (v) *Tg*–*Pb*A mice treated with sulfadiazine (SDZ) to inhibit cyst formation (Figure 1A) (Settles et al. 2014). All mice were sacrificed at 6.5 days post‐*Pb*A infection, except for survival studies.

### 2.6 RNA Extraction and Gene Expression by Real‐Time PCR

As described by Leleu et al. total RNA was isolated from whole brains using the NucleoSpin RNA kit (Macherey‐Nagel; 750,955,250) and cDNA synthesis was performed with the SuperScript VILO kit (Invitrogen; 11,754,250) (Leleu et al. 2022). From a mix of cDNA with primers (Table S1) (Eurogentec), RT‐qPCR was carried out with SYBR Green Master Mix (Applied Biosystems; 4,385,612) on a QuantStudio 12K Flex system (ThermoFisher) to quantify cytokine/chemokine expression and parasitemia. Gene expression was normalized to *Hprt1* (Hypoxanthine phosphoribosyltransferase 1 gene) and analyzed using the ΔΔCt method.

### 2.7 Histology of Brain Sections

As described by Hellani et al. optimal cutting temperature compound (OCT) was used to embed tissue of control and (co) infected mice (Hellani et al. 2024). Coronal brain sections were cut serially at 5 μm thickness, spread onto Super‐frost Plus slides (ThermoFisher, J1820AMNZ), and then allowed to dry vertically overnight at 37°C. To visualize parasites in cerebral parenchyma and to assess the degree of immune cell infiltration, vascular infiltration, and edema in meninges, brain sections were stained via hematoxylin and eosin (H&E). Slides were coverslipped using DPX mounting agent (Sigma‐Aldrich). Slides were finally examined under light microscopy at 60× magnifications, and images were taken using a digital camera mounted on the microscope.

### 2.8 T_2_W Images Sequence Magnetic Resonance Imaging (MRI)

T_2_ weighted (T_2_W) MRI sequences were performed on a Biospec 7.0 T/20 cm machine with a horizontal magnet (Bruker), as previously described by Dalko et al. (2016). At day 27.5, each group of animals was anesthetized with isoflurane and then placed in a dual‐coil small animal restrainer containing a volume coil for transmission and a surface quadratic coil for reception of signals. Coil‐to‐coil electromagnetic interaction was actively decoupled. Respiration rates and waveforms were continuously monitored via a force transducer. A feedback‐regulated circulating water pad was used to maintain the rectal temperature at 37°C ± 1°C. The acquisition of anatomic T_2_W images was done in axial and sagittal planes via the fast spin‐echo TurboRARE (rapid acquisition relaxation‐enhanced) pulse sequence, specifically configured (repetition time: 5000 ms; echo time: 77 ms; matrix size: 256 × 256 pixels; field of view: 20 × 20 mm; number of excitations: 4). Acquisition time was 10 min for each T_2_W sequence and choice of non‐overlapping 0.5 mm‐thick slices was done to acquire axial and sagittal images. Apparent diffusion coefficient (ADC) was measured to estimate diffusion rates.

### 2.9 Assessment of Brain Vascular Permeability

BBB integrity was assessed 6.5 days post‐*Pb*A infection using the Evans blue assay (Sigma‐Aldrich; E2129), as described by Keswani et al. (2020). Brains were perfused, incubated in formamide, and dye extravasation quantified spectrophotometrically at 620 nm. Using a standard curve, Evans blue concentration was calculated and expressed per g of brain tissue. Visually, blue coloration of the tissue was a sign of brain vascular permeability.

### 2.10 Isolation of Total Brain Cells

At day 27.5, brains were dissociated using a neural tissue dissociation kit (Miltenyi Biotec, 130‐093‐231), as described by Keswani et al. (2020). Then, cells were purified by Percoll (Cytiva; 11748498) gradient centrifugation, erythrocytes lysed with ammonium‐chloride‐potassium (ACK) buffer, and viability assessed by trypan blue exclusion (Gibco; 15250061).

### 2.11 Extra−/Intra‐Cellular Staining and Flow Cytometry

Cells were first treated with brefeldin A at 0.01 mg/mL (Sigma; B6542) for 4 h at 37°C to allow intracellular cytokine accumulation. After washing with 1× Phosphate‐Buffered Saline (PBS) (Gibco; 10010023), total brain cells were stained for surface and lineage markers for 30 min at 4°C in 1× PBS (Gibco; 10010023) containing 10% FBS (Dutscher; S181B‐500) using the antibodies listed in Table S2. Cells were then washed twice, fixed, and permeabilized using the CytoPerm‐CytoFix kit (BD Biosciences; AB_2869008) for 20 min at 4°C, and washed again.

Intracellular staining, as activation markers and cytokines and chemokines in glial cells and brain ILC2s, was subsequently performed by overnight incubation at 4°C with the appropriate antibody cocktail (Table S2) diluted in PBS containing 10% FBS (Dutscher; S181B‐500). After two washes, cells were either directly acquired or subjected to a final streptavidin staining step when required before resuspension in PBS containing 10% FBS. Data from 1 to 5 × 10^5^ events were acquired using the LSR Fortessa (Becton Dickinson). Compensation was performed using single‐stained controls. Data were analyzed using FlowJo software (version 10.9). Cell populations were identified according to the gating strategies shown in the corresponding figures and Supporting Information figure, and GFAP, MHC class I and II, CXCL‐10, TGF‐β, IL‐5 and IL‐13 expression was quantified by intracellular flow cytometry as normalized mean fluorescence intensity (MFI) within gated glial cells and ILC2s.

### 2.12 Confocal Microscopy of Brain Sections

Immunofluorescence staining was performed as described in (Hellani et al. 2024). Briefly, coronal brain sections (5 μm, OCT‐embedded; FisherScientific; 11912365), spread into Super‐frost Plus slides (ThermoFisher; J1820AMNZ), were fixed in 4% paraformaldehyde (PFA; Electron Microscopy Sciences; 15,713 R7G5), permeabilized with 0.1% Triton X‐100 (Sigma‐Aldrich; T8787), and blocked (30 min in 1×) PBS containing 5% FBS (Dutscher; S181B‐500) prior to immunofluorescence staining. Primary antibodies targeted GFAP (glial fibrillary acidic protein; 1/400; ThermoFisher; 53‐9892‐80), IL‐33 (1/200; R&D systems; AF3626), ST2 (1/200; Biolegend; 165302), and GATA3 (GATA‐binding protein 3; 1/100; Santa Cruz Biotechnology; sc‐268 AF594), followed by appropriate secondary antibodies (anti‐mouse IgG‐AF633 (1/200; Invitrogen; A21050); anti‐mouse IgG‐AF555 (1/200; Invitrogen; A31570)) and DAPI nuclear counterstain (Invitrogen; D1306). Images were acquired on a Zeiss LSM710 confocal microscope (ZEISS microscopy GmbH equipped with Airyscan super‐resolution (0.325 μm/pixel)).

### 2.13 Statistical Analysis

Each experiment was performed at least twice with three to ten animals per group. GraphPad Prism (Version 9; GraphPad software Inc.) was used for statistical analyses, with data expressed as a mean or median ± standard error of the mean (SEM). Comparisons between two groups were performed using the unpaired Student's *t*‐test. For comparisons involving more than two groups, the Kruskal–Wallis test followed by Dunn's multiple comparisons test was used. Comparison of survival was performed using the Log‐Rank (Mantel Cox) test. Hierarchical clustering of the samples and cytokines/chemokines quantification were generated based on the Euclidean distance, using Perseus software (Max Planck Institute of Biochemestry) and expressed on heatmap after *Y* values transformation as *Y* = Log2(*Y*) with Prism. *p* values indicate statistical significance as *p < 0.05, **p < 0.01, ***p < 0.001, and ****p < 0.0001.

## 3 Results

### 3.1 Cerebral Persistence of *T. gondii* Is Required for Protection Against ECM Independently of *P. berghei* Parasite Burden

To investigate the mechanisms underlying the protection against experimental cerebral malaria (ECM) induced by chronic *Toxoplasma gondii* (*Tg*) infection, we adapted the model previously described by Settles et al. (2014). C57BL/6 mice were first infected with type II *Tg* (Pru strain) tachyzoites and challenged 21 days later with 10^6^ blood‐stage parasites of *Pb*A clone 1.4 (Figure 1A). This 21‐day interval allows the establishment of chronic cerebral toxoplasmosis characterized by the presence of brain bradyzoite cysts before *Pb*A infection. *Pb*A infection of C57BL/6 mice reproducibly induces experimental cerebral malaria (ECM), with neurological symptoms and mortality occurring within the classical ECM window of 6–10 days post‐infection. Mono‐infected *Tg*, mono‐infected *Pb*A, and non‐infected control mice were included throughout the study. To determine whether chronic cerebral *Tg* infection was required for protection, sulfadiazine (SDZ) was administered from day 3 to day 17 after *Tg* infection to eliminate tachyzoites before brain colonization while preserving the early systemic inflammatory response. In parallel, a *Pb*A‐infected group also received SDZ treatment to determine whether the drug itself affected ECM development (Figure 1A). As expected, *Pb*A‐infected mice developed ECM within the classical neurological window of 6–10 days post‐infection (pink window in Figure 1B). SDZ treatment alone did not modify disease progression, as *Pb*A‐ and SDZ + *Pb*A‐infected mice displayed comparable survival kinetics. In contrast, chronically *Tg*‐infected mice were fully protected from ECM following *Pb*A challenge and died later from hyperparasitemia. Importantly, SDZ treatment completely abolished the protection, demonstrating that preventing the establishment of chronic cerebral *Tg* infection abrogates resistance to ECM (Figure 1B).

We next assessed chronic cerebral *Tg* infection by quantifying the expression of BAG‐1, a bradyzoite‐specific marker, by RT‐qPCR. BAG‐1 transcripts were readily detected in the brains of *Tg*‐infected and coinfected mice but were undetectable in control mice and in both SDZ‐treated groups, confirming that SDZ efficiently prevented the establishment of chronic cerebral *Tg* infection (Figure 1C, left). Although not statistically significant, coinfected mice exhibited a trend toward lower cerebral BAG‐1 expression than *Tg*‐infected mice. Consistent with these findings, cyst enumeration following immunofluorescent detection of the cyst wall antigen on brain sections revealed numerous tissue cysts in *Tg*‐infected and coinfected mice, whereas no cysts were detected in SDZ‐treated animals (Figure 1C, right). Coinfected mice also tended to harbor fewer cerebral cysts than *Tg*‐infected mice, although this difference did not reach statistical significance.

To determine whether protection resulted from altered *Pb*A replication, we next quantified cerebral *Pb*A burden by the measure of *Pb18s* (18S ribosomal RNA gene of *Plasmodium berghei*) transcript levels by RT‐qPCR and peripheral parasitemia by Giemsa‐stained blood smears. Comparable cerebral *Pb*A parasite loads were detected in *Pb*A‐infected, coinfected, SDZ + *Pb*A, and SDZ + coinfected mice, whereas no *Pb*A signal was detected in non‐infected control mice (Figure 1D, left). Similarly, peripheral parasitemia did not differ significantly among the four *Pb*A‐infected groups (Figure 1D, right), indicating that neither chronic *Tg* infection nor SDZ treatment altered *Pb*A replication.

Histological analysis of brains from *Tg*–*Pb*A coinfected mice revealed the coexistence of *Tg* tissue cysts within the brain parenchyma and *Pb*A‐infected erythrocytes sequestered in cerebral microvessels (Figure 1E). These observations demonstrate that, despite complete protection from ECM, cerebral sequestration of infected erythrocytes persists in coinfected animals, indicating that protection is not mediated by reduced vascular parasite accumulation. Collectively, these findings demonstrate that protection against ECM requires the establishment of chronic cerebral *T. gondii* infection and tissue cyst formation but occurs independently of peripheral parasitemia, cerebral *Pb*A burden, and cerebral sequestration of infected erythrocytes. These observations support the hypothesis that chronic *T. gondii* infection promotes a host‐mediated disease‐tolerance mechanism rather than enhanced control of *P. berghei* replication.

### 3.2 Coinfection Preserves BBB Integrity and Limits *Pb*A‐Induced Cerebral Edema

We assessed physiological and morphological changes in the CNS of *Pb*A‐infected mice during ECM, as well as in *Tg*‐only and *Tg*–*Pb*A coinfected mice, using MRI. T_2_‐weighted (T_2_W) images revealed pronounced alterations in the corpus callosum of *Pb*A‐infected mice (yellow box in control images), consistent with edema and hyperintense lesions indicative of structural damage typically associated with ECM (Figure 2A). Volumetric analysis confirmed a significant increase in corpus callosum volume in *Pb*A‐infected mice compared to non‐infected controls and *Tg*‐infected groups (Figure 2B). In contrast, both *Tg* groups exhibited preservation of corpus callosum integrity, with volumes comparable to controls (Figure 2A,B). Analysis of ventricular morphology showed distinct patterns in the third ventricle (yellow arrows): *Tg*‐only and *Tg*–*Pb*A mice exhibited increased visibility primarily in the upper portion, whereas ECM^+^ mice displayed enlargement predominantly in the lower portion (Figure 2A), reflecting differential structural remodeling and highlighting distinct neuropathological signatures between *Tg*‐ and *Pb*A‐driven cerebral alterations. In addition, MRI assessment of the olfactory bulbs (yellow ellipse in Figure 2A) demonstrated marked volumetric expansion across all infected groups compared to uninfected controls (Figure 2B). Notably, co‐ and *Tg*‐only infected mouse brain presented approximately two‐fold larger olfactory bulbs than ECM^+^ group. No significant difference was observed between *Tg*‐only and *Tg*–*Pb*A groups, indicating that *Tg* infection, independently of *Pb*A coinfection, drives this pronounced olfactory bulb hypertrophy. Diffusion‐weighted imaging revealed reduced ADC values in cortical regions of *Tg*‐only and coinfected mice compared to *Pb*A group, consistent with reduced neuroinflammatory activity compared to that observed during ECM (Figure 2C). Consistently, BBB permeability assessed by Evans blue dye infiltration was increased in ECM^+^ mice but remained unaltered in *Tg*–*Pb*A coinfected mice, indicating preservation of BBB integrity during coinfection (Figure 2D). Finally, histological analysis confirmed the MRI observations. *Pb*A‐infected mice displayed pronounced parenchymal immune cell infiltration and vasculitis, whereas these features were markedly reduced in *Tg*–*Pb*A coinfected mice. All infected groups exhibited meningeal immune infiltration and extracellular edema, with meningeal thickening present in both *Pb*A‐ and *Tg*‐infected animals compared to controls. Notably, parenchymal inflammation and vasculitis were attenuated in coinfected mice relative to *Tg*‐only mice (Figure 2E). Collectively, these findings show that protection against *Pb*A‐induced neuropathology during *Tg* coinfection is associated with preserved BBB integrity and reduced neuroinflammatory damage, consistent with a disease tolerance mechanism.

**FIGURE 2:**
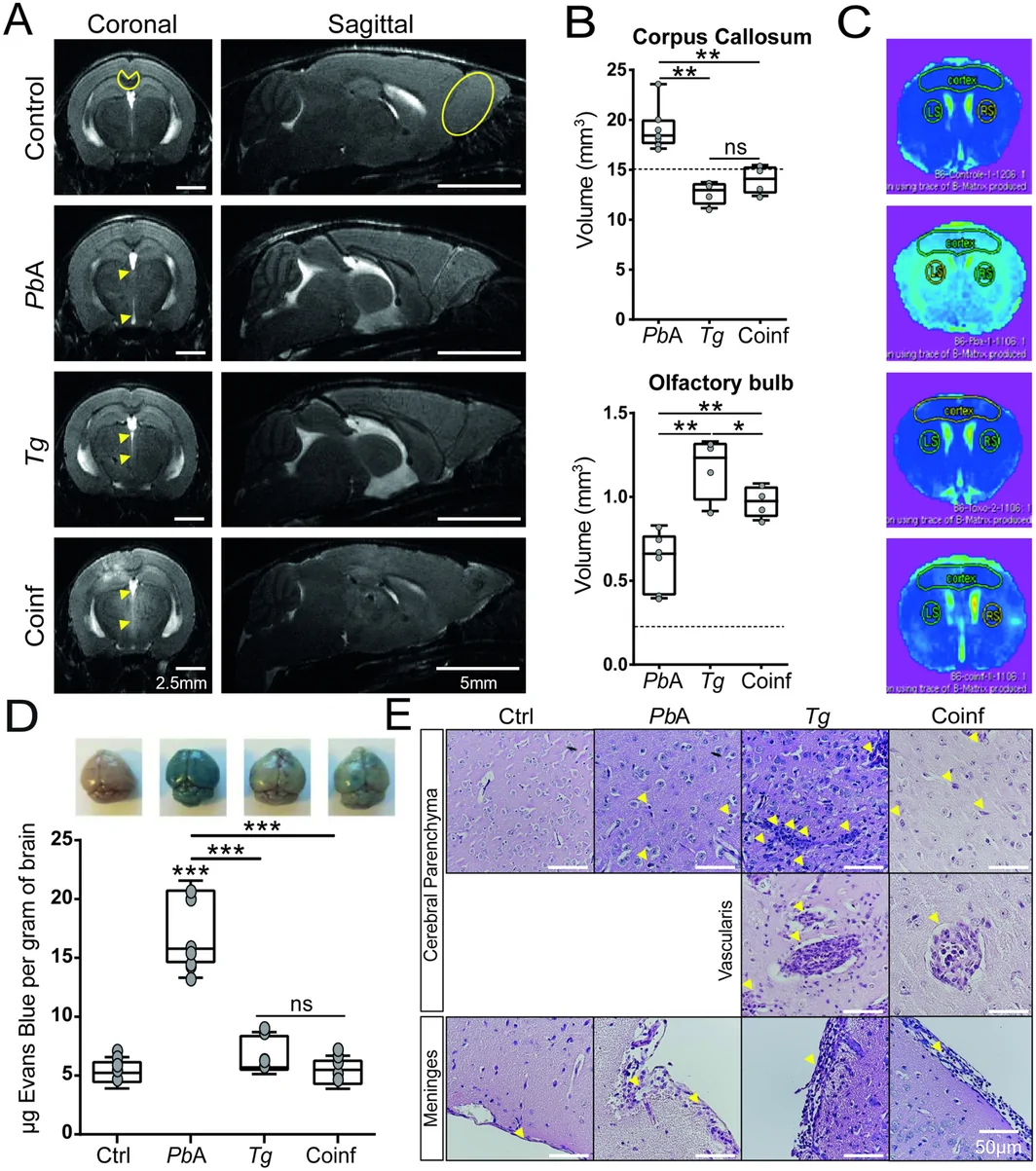
Coinfected is associated with reduced brain edema and preserved BBB integrity. (A, B) Coronal (scale bar = 2.5 mm) and sagittal (scale bar = 5 mm) T_2_‐weighted MRI images of control, ECM^+^, *Tg*‐only, and *Tg*–*Pb*A mice. Yellow boxes indicate hyperintense regions in the corpus callosum, ellipses mark olfactory bulbs, and arrows indicate third ventricle enlargement. (C) ADC maps indicating reduced tissue alterations in coinfected mice compared to ECM^+^ mice. (D) Evans blue assay showing increased BBB permeability in *Pb*A‐infected mice but not in coinfected mice. (E) Histological analysis of brain sections stained with hematoxylin and eosin (H&E). Coinfected mice display reduced parenchymal immune infiltration and vasculitis (yellow arrows) compared to *Tg*‐only mice. Meningeal thickening is observed in all infected groups. Scale bar = 50 μm. Data (*n* = 5–8, three independent experiments) are presented as mean ± SEM. Statistical analysis was performed using the Kruskal–Wallis test followed by Dunn's multiple‐comparison test or Student's *t*‐test. ns, not significant; *p < 0.05; **p < 0.01; ***p < 0.001.

### 3.3 Chronic Cerebral *Tg* Infection Is Associated With a Regulated Neuroinflammatory Transcriptional Profile Characterized by TGF‐β and IL‐33/ST2 Expression

To investigate the molecular basis of the regulated neuroimmune environment associated with *Tg*‐mediated protection against ECM, we quantified cytokines and chemokines in the brain of control, *Pb*A‐, *Tg*‐, and *Tg*–*Pb*A‐infected mice by RT‐qPCR (Figure 3A). Gene expression analysis identified three distinct transcriptional signatures corresponding to control, ECM, and chronic *Tg*‐associated neuroinflammatory states. Both *Pb*A and *Tg* infection induced the expression of pro‐inflammatory mediators such as *Cxcl‐10*, *Ccl‐2*, *Il‐6*, and *Tnf‐α*, compared with control mice. *Il‐10* expression was also increased in infected brains. However, chronic *Tg* infection and coinfection were distinguished by the concomitant upregulation of *Tgf‐β*, *Il‐33a* and its receptor *St2*, whereas expression of the inducible *Il‐33b* isoform remained low (Figure 3A). Although *Cxcl‐10* expression increased in all infected groups, its induction remained substantially lower in *Tg*‐infected and coinfected brains than in *Pb*A‐infected mice. In contrast, chronic *Tg* infection was associated with a marked increase in *Tgf‐β*, *Il‐33a*, and *St2* expression, highlighting the emergence of a distinct neuroimmune transcriptional profile (Figure 3B). Collectively, these data indicate that chronic cerebral *Tg* infection is associated with a regulated neuroinflammatory profile characterized by enhanced TGF‐β and IL‐33/ST2 signaling, supporting the establishment of a regulated neuroimmune environment associated with disease tolerance during ECM.

**FIGURE 3:**
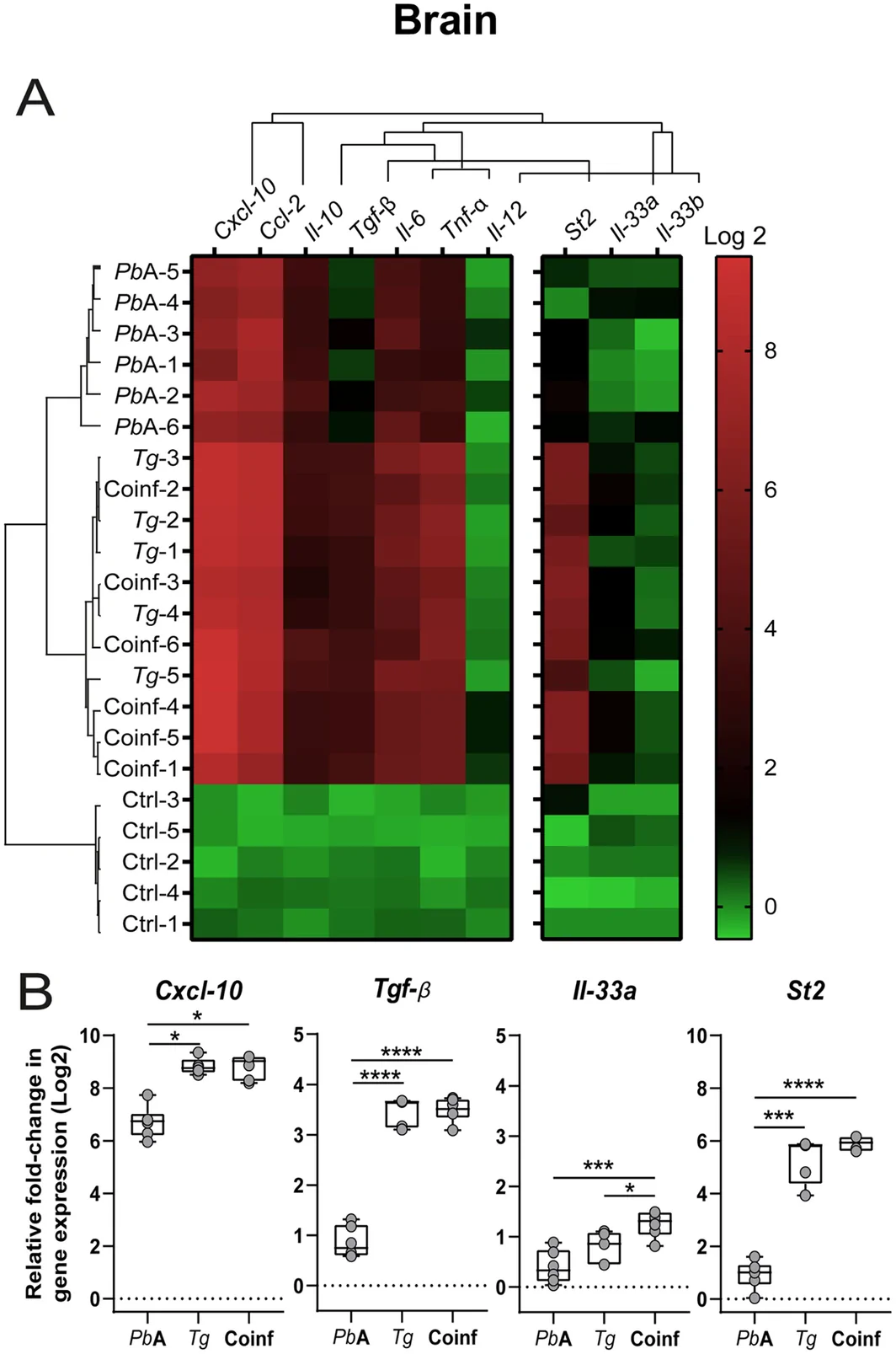
Chronic cerebral *Tg* infection is associated with a regulated neuroinflammatory transcriptional profile characterized by TGF‐β and IL‐33/ST2 expression. (A) Relative mRNA expression of pro‐inflammatory and immunoregulatory cytokines and chemokines in the brains of control, *Pb*A‐, *Tg*‐, and *Tg*–*Pb*A‐infected mice, quantified by RT‐qPCR. Heatmap values are presented as log_2_ fold change relative to control mice. (B) Relative expression of selected genes (*Cxcl‐10*, *Tgf‐β*, *Il‐33a*, and *St2*) highlighting the distinct neuroinflammatory transcriptional profiles associated with *Pb*A infection, chronic *Tg* infection, and *Tg*–*Pb*A coinfection. Gene expression levels were normalized to control mice and are presented as relative fold change. Data are presented as box‐and‐whisker plots showing the median (center line), the 25th–75th percentiles (box), and the minimum and maximum values (whiskers), with all individual data points displayed. Results are pooled from three independent experiments (*n* = 5–15 mice per experimental group). Statistical analyses were performed using the Kruskal–Wallis test followed by Dunn's multiple‐comparison test. *p < 0.05; ***p < 0.001; ****p < 0.0001.

### 3.4 Coinfection Promotes Astrocyte Remodeling Associated With an Intermediate Inflammatory Phenotype and IL‐33/ST2 Expression

To investigate whether astrocytes contribute to the regulated neuroimmune environment associated with protection against ECM, we first quantified total brain cellularity by flow cytometry. All infected groups displayed a significant increase in total brain cell numbers compared with uninfected controls (Figure 4A). While *Pb*A infection induced only a modest increase, chronic *T. gondii* infection resulted in a marked expansion of brain cellularity, which was also observed during coinfection, although to a slightly lesser extent than in *Tg*‐infected mice. Astrocytes were identified as GLAST^+^CD11b^−^ cells (Figure 4B). Consistent with the increase in total brain cellularity, the absolute number of GLAST^+^ astrocytes was significantly higher in *Tg*‐infected and coinfected mice than in control and *Pb*A‐infected animals. Analysis of astrocyte frequencies confirmed these observations. Whereas GLAST^+^ astrocytes represented approximately 18%–20% of total brain cells in control and *Pb*A‐infected mice, their proportion increased to approximately 30% in *Tg*‐infected and coinfected animals, indicating that chronic *Tg* infection promotes astrocyte expansion independently of secondary *Pb*A infection (Figure 4B).

**FIGURE 4:**
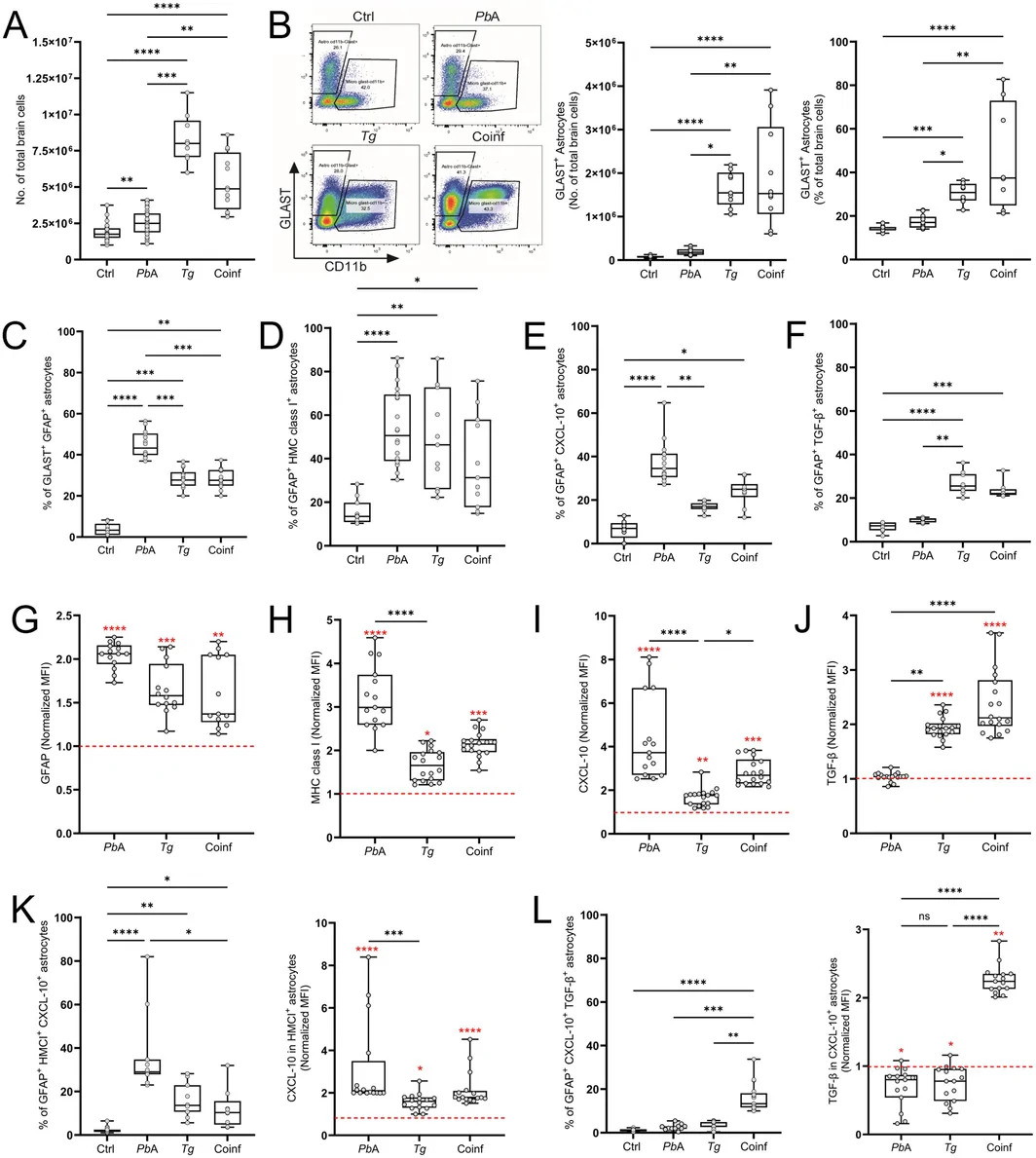
Coinfection promotes astrocyte remodeling associated with an intermediate inflammatory phenotype. (A) Total number of brain cells determined by *flow cytometry* in control, *Pb*A‐infected, *T. gondii*‐infected (*Tg*) and coinfected mice. (B) Representative *flow cytometry* gating strategy used to identify astrocytes as GLAST^+^CD11b^−^ cells. Absolute number and frequency (%) of GLAST^+^ astrocytes among total brain cells of the different experimental groups. (C) Frequency (%) of GFAP^+^ astrocytes within the GLAST^+^ population. (D–F) Frequency (%) of GFAP^+^ astrocytes expressing MHC‐I (D), CXCL‐10 (E) or TGF‐β (F). (G–J) Normalized MFI of GFAP (G), MHC‐I (H), CXCL‐10 (I) and TGF‐β (J) in GFAP^+^ astrocytes. MFI values were normalized to the MFI of the control group. (K) Frequency (%) of GFAP^+^MHC‐I^+^CXCL‐10^+^ astrocytes (left) and normalized CXCL‐10 MFI within MHC‐I^+^ astrocytes (right). (L) Frequency (%) of GFAP^+^CXCL‐10^+^TGF‐β^+^ astrocytes (left) and normalized TGF‐β MFI within CXCL‐10^+^ astrocytes (right). All quantitative data were obtained by flow cytometry. Data are presented as box‐and‐whisker plots showing the median, interquartile range, minimum and maximum values, with individual data points displayed. Results are pooled from three independent experiments (*n* = 10–20 mice per experimental group). Statistical analyses were performed using the Kruskal–Wallis test followed by Dunn's multiple‐comparison test. *p < 0.05; **p < 0.01; ***p < 0.001; ****p < 0.0001.

We next evaluated astrocyte activation by analyzing GFAP expression within the GLAST^+^ population. The proportion of GFAP^+^ astrocytes increased significantly in all infected groups compared with controls, with the highest frequencies observed in *Pb*A‐infected mice. *Tg*‐infected and coinfected animals exhibited intermediate but comparable levels of astrocyte activation (Figure 4C). To further characterize the inflammatory profile of reactive astrocytes, we analyzed the expression of MHC‐I, CXCL‐10, and TGF‐β. The proportion of GFAP^+^MHC class I^+^ astrocytes increased significantly in all infected groups relative to controls without significant differences between infected groups, although a tendency toward lower frequencies was observed in *Tg*‐infected and coinfected mice (Figure 4D). Likewise, the proportion of GFAP^+^CXCL‐10^+^ astrocytes increased following infection, with *Pb*A‐infected mice displaying the highest frequencies, whereas *Tg*‐infected and coinfected mice exhibited lower but comparable levels (Figure 4E). In contrast, GFAP^+^TGF‐β^+^ astrocytes were selectively enriched in *Tg*‐infected and coinfected mice, while remaining unchanged in *Pb*A‐infected animals (Figure 4F). To determine whether these phenotypic differences were accompanied by changes in marker expression, we next quantified the normalized mean fluorescence intensity (MFI) of GFAP, MHC‐I, CXCL‐10, and TGF‐β (Figure 4G–J). GFAP, MHC‐I, and CXCL‐10 expression levels were significantly increased in all infected groups compared with controls, with *Pb*A‐infected mice consistently displaying the highest expression levels (Figure 4G–I). Conversely, TGF‐β expression was selectively increased in *Tg*‐infected and coinfected mice but not in *Pb*A‐infected animals, further supporting the establishment of an astrocyte phenotype associated with chronic *Tg* infection (Figure 4J).

Finally, we investigated whether reactive astrocytes simultaneously expressed pro‐inflammatory and immunoregulatory markers. The proportion of GFAP^+^MHC‐I^+^CXCL‐10^+^ astrocytes increased significantly in all infected groups, with the highest frequencies observed in *Pb*A‐infected mice. Consistently, CXCL‐10 expression within MHC‐I^+^ astrocytes was significantly higher in *Pb*A‐infected animals than in *Tg*‐infected or coinfected mice (Figure 4K). In contrast, GFAP^+^CXCL‐10^+^TGF‐β^+^ astrocytes were significantly enriched only in coinfected mice. Moreover, TGF‐β expression within CXCL‐10^+^ astrocytes was selectively increased during coinfection (Figure 4L). Together, these observations identify a population of reactive astrocytes co‐expressing CXCL‐10 and TGF‐β that is specifically associated with coinfection, consistent with the establishment of an intermediate inflammatory astrocyte phenotype.

Having identified this distinct astrocyte population during coinfection, we next investigated whether these reactive astrocytes expressed IL‐33, an alarmin implicated in CNS immune regulation. In WT mice, the proportion of IL‐33^+^ brain cells increased significantly in all infected groups compared with controls, with a modest increase following *Pb*A infection and a much stronger increase during chronic *Tg* infection and coinfection. As expected, IL‐33 expression was undetectable in IL‐33‐deficient (IL33^−/−^) mice, confirming the specificity of the staining (Figure 5A). We next analyzed IL‐33 expression within astrocytes. The proportion of GFAP^+^IL‐33^+^ astrocytes, expressed as a percentage of total brain cells, increased significantly in all infected WT mice, with the highest frequencies observed during *Tg* infection and coinfection. In contrast, no GFAP^+^IL‐33^+^ astrocytes were detected in IL‐33‐deficient mice (Figure 5B). These results identify reactive astrocytes as one of the major IL‐33‐expressing cell populations associated with chronic *Tg* infection and coinfection.

**FIGURE 5:**
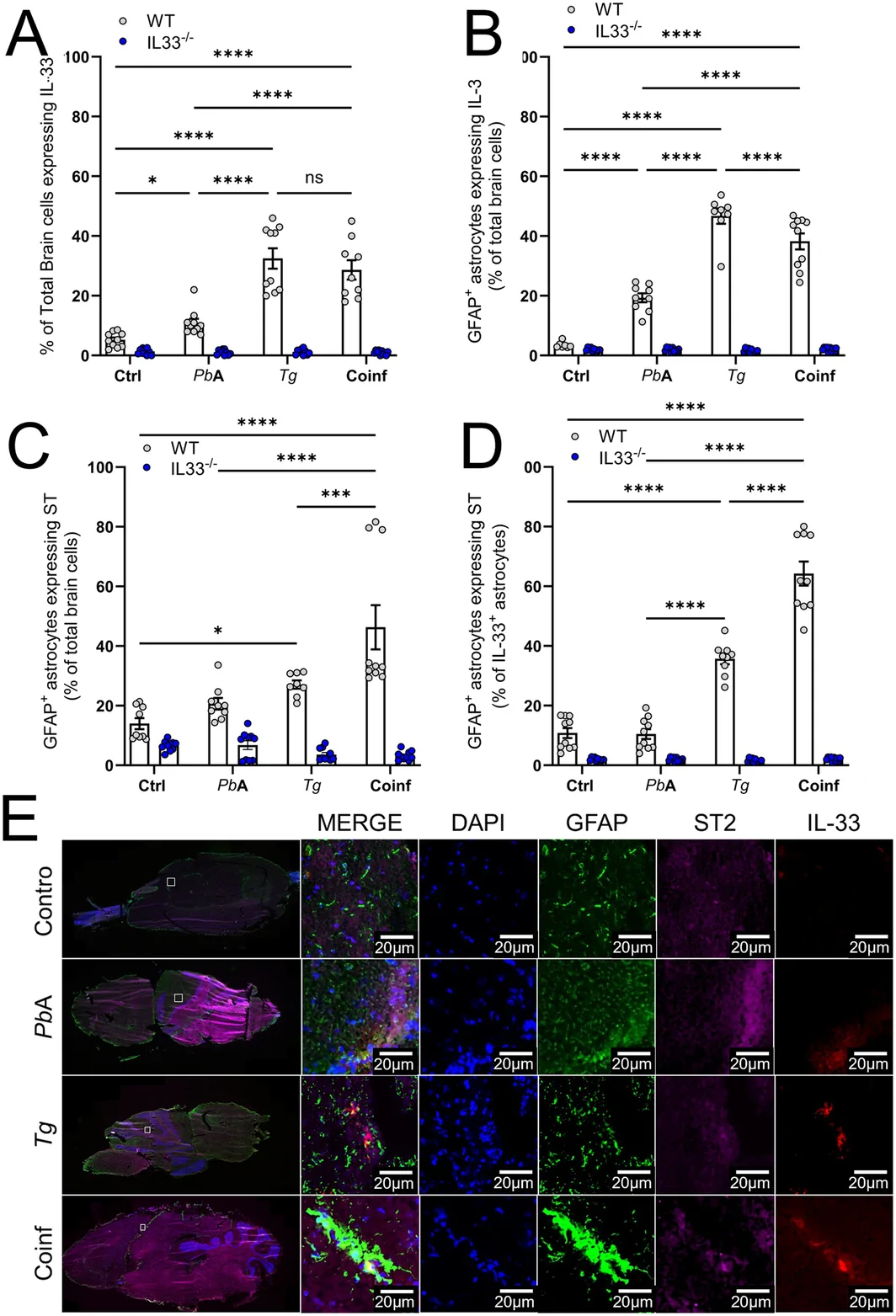
Reactive astrocytes represent one of the major IL‐33‐expressing cell populations during chronic *T. gondii* infection and coinfection. (A) Frequency (%) of IL‐33^+^ cells among total brain cells determined by *flow cytometry* in wild‐type (WT) and IL‐33‐deficient (IL‐33^−/−^) mice. (B, C) Frequency (%) of GFAP^+^IL‐33^+^ (B) and GFAP^+^ST2^+^ (C) astrocytes among total brain cells in WT and IL‐33^−/−^ mice. (D) Frequency (%) of ST2^+^ astrocytes within the IL‐33^+^ astrocyte population in WT and IL‐33^−/−^ mice. (E) Representative confocal images of brain sections from WT control, *Pb*A‐infected, *Tg*‐infected and coinfected mice following immunofluorescence stained for GFAP (green), IL‐33 (red), ST2 (magenta) with DAPI nuclear counterstaining (blue). Merged and single‐channel images illustrate the colocalization of IL‐33 and ST2 within GFAP^+^ astrocytes, particularly in *Tg*‐infected and coinfected brains. Scale bar = 20 μm. All quantitative data were obtained by flow cytometry. Data are presented as box‐and‐whisker plots showing the median (center line), the 25th and 75th percentiles (box), and the minimum and maximum values (whiskers), with individual data points displayed. Results are pooled from three independent experiments (*n* = 10–20 mice per experimental group). Statistical analyses were performed using the Kruskal–Wallis test followed by Dunn's multiple‐comparison test. *p < 0.05; **p < 0.01; ***p < 0.001; ****p < 0.0001.

Because IL‐33 signals through its receptor ST2, we next assessed ST2 expression in astrocytes. The proportion of GFAP^+^ST2^+^ astrocytes increased significantly in *Tg*‐infected and coinfected mice but not following *Pb*A infection alone. The highest frequencies were consistently observed in coinfected animals. In IL‐33‐deficient mice, only low frequencies of GFAP^+^ST2^+^ astrocytes were detected in all experimental groups (Figure 5C). To determine whether IL‐33‐producing astrocytes also expressed their cognate receptor, we analyzed ST2^+^ astrocytes within the IL‐33^+^ astrocyte population. Whereas no double‐positive astrocytes were detected in IL‐33‐deficient mice, WT animals exhibited a marked increase in IL‐33^+^ST2^+^ astrocytes during chronic *Tg* infection, with the highest frequencies observed in coinfected mice (Figure 5D). Confocal microscopy further confirmed these observations. Whereas control brains displayed little ST2 staining and weak IL‐33 expression, *Tg* infection—and even more prominently coinfection—resulted in strong IL‐33 expression together with increased ST2 staining within GFAP‐positive astrocytes, demonstrating extensive colocalization of IL‐33 and ST2 in reactive astrocytes (Figure 5E).

Collectively, these findings indicate that chronic *T. gondii* infection establishes an IL‐33/ST2‐expressing astrocyte compartment that is further reshaped following *Pb*A superinfection. This remodeling is associated with the emergence of reactive astrocytes co‐expressing CXCL‐10 and TGF‐β, consistent with an intermediate inflammatory phenotype observed specifically during coinfection. Reactive astrocytes therefore represent one of the major IL‐33‐expressing cell populations associated with the regulated neuroimmune environment in coinfected mice.

### 3.5 Reactive Astrocyte‐Associated IL‐33 Signaling Contributes to the Protection Against ECM and Is Associated Cerebral ILC2 Recruitment During *Tg–PbA* Coinfection

Having identified reactive astrocytes as one of the major IL‐33‐expressing cell populations during chronic *T. gondii* infection and coinfection, we next investigated whether IL‐33 contributed to the protective phenotype observed during *Tg*–*Pb*A coinfection. Survival was therefore monitored in IL‐33‐deficient mice following *Tg*–*Pb*A coinfection. In contrast to the complete protection observed in wild‐type coinfected mice, IL‐33‐deficient coinfected mice displayed an intermediate survival phenotype. Approximately 50%–60% of the animals succumbed around 12 days post‐*Pb*A infection, corresponding to a delayed ECM phase, whereas the remaining mice survived beyond the neurological window and subsequently died from hyperparasitemia (Figure 6A). These findings indicate that IL‐33 contributes substantially to the protection conferred by chronic *T. gondii* infection against ECM, although its absence does not completely abolish this protective effect.

**FIGURE 6:**
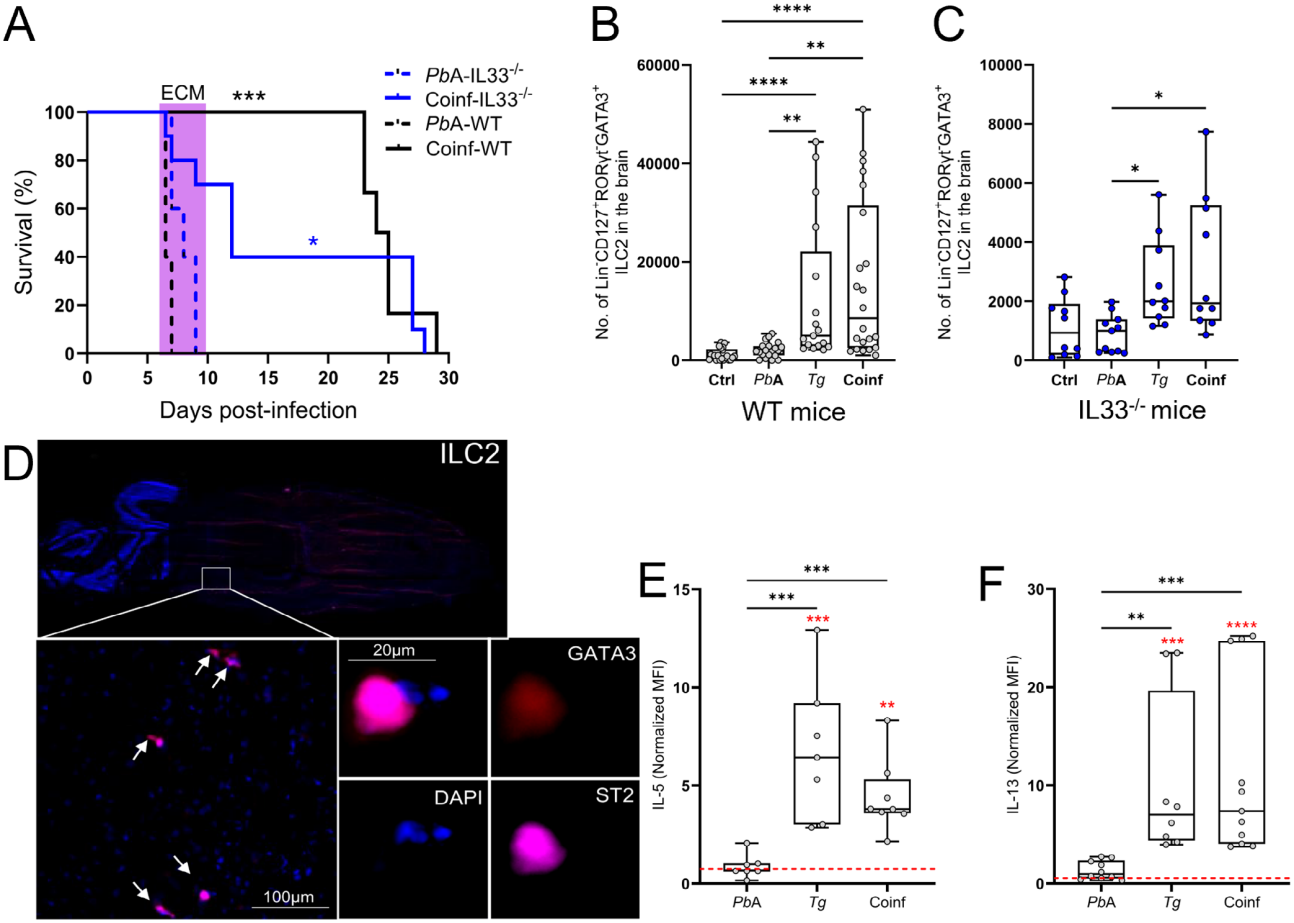
IL‐33 contributes to protection against ECM and cerebral ILC2 accumulation during *Tg*–*Pb*A coinfection. (A) Kaplan–Meier survival curves of wild‐type (WT) and IL‐33^−/−^ mice infected with *Pb*A alone or coinfected with *Tg* and *Pb*A. WT and IL‐33^−/−^ mice infected with *Pb*A alone succumbed during the classical ECM window (6–10 days post‐*Pb*A infection, highlighted in violet). In contrast, WT coinfected mice were protected during the ECM window and subsequently died from hyperparasitemia. IL‐33^−/−^ coinfected mice displayed an intermediate survival phenotype, with approximately 50%–60% of the animals dying around day 12 post‐*Pb*A infection, whereas the remaining mice survived beyond the neurological window and later died from hyperparasitemia. These data indicate that IL‐33 contributes to, but is not solely responsible for, the protective phenotype observed during chronic *Tg*–*Pb*A coinfection. Data represent *n* = 15 mice per group from three independent experiments. Survival curves were compared using the Mantel–Cox (log‐rank) test. (B, C) Absolute numbers of cerebral ILC2s quantified by *flow cytometry* in wild‐type (WT) (B) and IL‐33‐deficient (IL33^−/−^) (C) control, *Pb*A‐infected, *T. gondii*‐infected (*Tg*) and *Tg*–*Pb*A coinfected mice. (D) Representative confocal images of brain sections from WT *Tg*–*Pb*A coinfected mice following immunofluorescence staining for GATA3 (red), ST2 (magenta) and nuclei (DAPI, blue), illustrating the presence of GATA3^+^ST2^+^ cells consistent with ILC2s in the brain. (E, F) Normalized MFI of IL‐5 (E) and IL‐13 (F) in cerebral ILC2s from WT mice. All quantitative data were obtained by *flow cytometry*. Data are presented as box‐and‐whisker plots showing the median (center line), the 25th–75th percentiles (box), and the minimum and maximum values (whiskers), with individual data points displayed. Results are pooled from three independent experiments (*n* = 6–20 mice per experimental group). Statistical analyses were performed using the Kruskal–Wallis test followed by Dunn's multiple‐comparison test. *p < 0.05; **p < 0.01; ***p < 0.001; ****p < 0.0001.

Given the established role of IL‐33 in the recruitment and activation of ILC2s, we next investigated whether the altered disease outcome observed in IL‐33‐deficient mice was associated with changes in the cerebral ILC2 compartment. Brain ILC2s were identified by flow cytometry as Lineage^−^CD127^+^RORγt^−^GATA3^+^ cells (Figure S1). Flow cytometric analysis revealed a marked accumulation of cerebral ILC2s in WT mice during chronic *Tg* infection and coinfection. The absolute number of brain ILC2s was significantly increased in both *Tg*‐infected and coinfected mice compared with uninfected controls, whereas *Pb*A infection alone induced only a modest, non‐significant increase. No significant difference was observed between *Tg*‐infected and coinfected mice, indicating that chronic *Tg* infection promotes the accumulation of ILC2s within the brain (Figure 6B). To determine whether IL‐33 contributes to this response, we next quantified cerebral ILC2s in IL‐33‐deficient mice. Although *Tg* infection and coinfection still resulted in a significant increase in brain ILC2 numbers compared with uninfected IL‐33‐deficient mice, the magnitude of this response was markedly reduced compared with WT animals (Figure 6C). Indeed, IL‐33‐deficient mice displayed approximately four‐fold fewer cerebral ILC2s than their WT counterparts, indicating that IL‐33 represents an important, although not exclusive, regulator of cerebral ILC2 accumulation (Figure 6B,C). Representative confocal microscopy confirmed the presence of GATA3^+^ST2^+^ ILC2s within the brains of coinfected WT mice, supporting the flow cytometric observations (Figure 6D). We next assessed the activation status of infiltrating ILC2s by intracellular flow cytometry, quantifying the expression of the canonical type 2 cytokines IL‐5 and IL‐13. The normalized MFI of both IL‐5 and IL‐13 was significantly increased in ILC2s isolated from *Tg*‐infected and coinfected WT mice compared with control and *Pb*A‐infected animals (Figure 6E,F). IL‐13 expression was consistently higher than IL‐5 during coinfection, suggesting that cerebral ILC2s acquire an activated type 2 phenotype in response to chronic *T. gondii* infection. Collectively, these findings identify IL‐33 as an important component of the protective response established during chronic *T. gondii* infection. The partial loss of protection observed in IL‐33‐deficient mice was accompanied by a marked reduction in cerebral ILC2 accumulation, while the ILC2s recruited to the brains of WT mice displayed increased expression of IL‐5 and IL‐13. Residual ILC2 accumulation in the absence of IL‐33 further indicates that additional IL‐33‐independent mechanisms contribute to their recruitment. Together, these data support an association between reactive astrocyte‐derived IL‐33, cerebral ILC2 accumulation and protection from ECM during *Tg*–*Pb*A coinfection.

### 3.6 Differential Activation of Brain‐Resident Microglia and Infiltrating Monocytes During *Tg*–PbA Coinfection

To investigate the impact of *Pb*A, *Tg* and coinfection on brain‐resident myeloid cells, microglia were identified by flow cytometry as CD11b^+^CD45^low^ cells. The absolute number of microglia was significantly increased in *Tg*‐infected and coinfected mice compared with uninfected controls, whereas coinfected mice exhibited significantly fewer microglia than *Tg*‐infected animals. Likewise, the proportion of microglia among total brain cells increased in all infected groups, with the most pronounced increase observed following *Tg* infection (Figure 7A). Phenotypic characterization revealed marked microglial activation following infection. The frequencies of CD86^+^ and CD16/32^+^ microglia were significantly increased in all infected groups compared with controls, indicating enhanced activation irrespective of the infecting pathogen (Figure 7B,C). In contrast, MHC class II expression was significantly increased only in coinfected mice, suggesting that coinfection promotes a distinct activation state associated with enhanced antigen‐presenting capacity (Figure 7D). Analysis of additional activation markers showed that the proportion of CD206^+^ microglia was significantly reduced only in *Tg*‐infected mice, whereas no significant changes were observed in *Pb*A‐ or coinfected animals (Figure 7E). Conversely, MHC class I expression was significantly increased in all infected groups compared with controls, indicating a generalized enhancement of antigen presentation during infection (Figure 7F).

**FIGURE 7:**
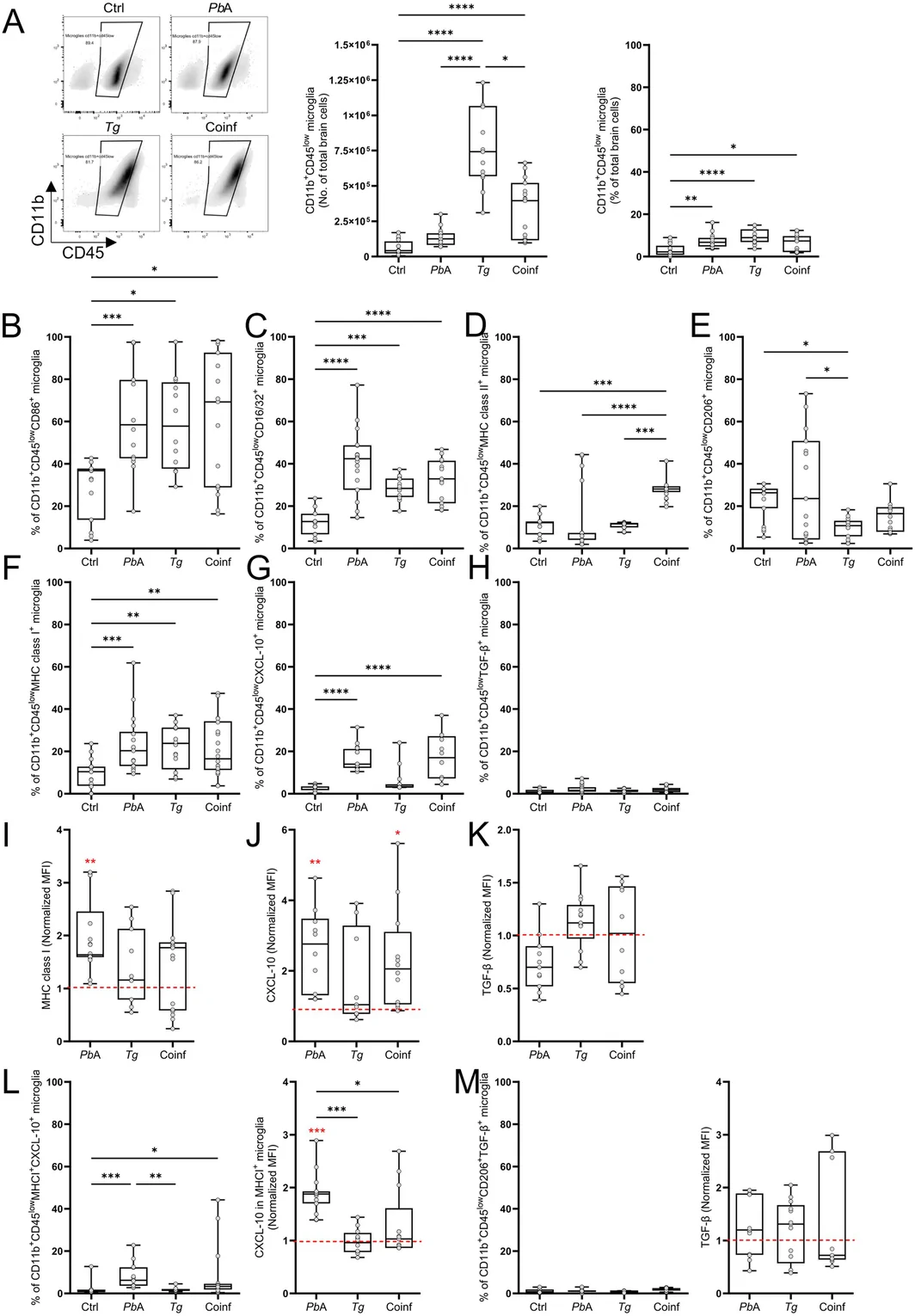
Differential activation of brain microglia during *Pb*A, *Tg* and coinfection. (A) Representative gating strategy used to identify brain microglia (CD11b^+^CD45^low^), together with the absolute number and frequency of microglia among total brain cells. (B–H) Frequency of CD86^+^ (B), CD16/32^+^ (C), MHC class II^+^ (D), CD206^+^ (E), MHC class I^+^ (F), CXCL‐10^+^ (G), and TGF‐β^+^ (H) microglia expressed as a percentage of total microglia. (I–K) Normalized MFI of MHC class I (I), CXCL‐10 (J), and TGF‐β (K) in microglia. MFI values were normalized to the mean value of the uninfected control group within each independent experiment (control = 1). (L) Frequency of MHC class I^+^CXCL10^+^ microglia expressed as a percentage of total microglia, together with normalized CXCL‐10 MFI within MHC class I^+^ microglia. (M) Frequency of CD206^+^TGF‐β^+^ microglia expressed as a percentage of total microglia, together with normalized TGF‐β MFI within CD206^+^ microglia. Data are presented as box‐and‐whisker plots showing the median (center line), the 25th–75th percentiles (box), and the minimum and maximum values (whiskers), with individual data points displayed (*n* = 8–20 mice pooled from three independent experiments). Statistical analyses were performed using the Kruskal–Wallis test followed by Dunn's multiple‐comparison test. *p < 0.05; **p < 0.01; ***p < 0.001; ****p < 0.0001.

To further characterize the inflammatory profile of microglia, intracellular CXCL10 and TGFβ expression were evaluated. The proportion of CXCL10^+^ microglia was significantly increased in *Pb*A‐ and coinfected mice compared with controls, whereas no significant increase was detected in *Tg*‐infected mice (Figure 7G). In contrast, the proportion of TGFβ^+^ microglia remained low (approximately 1%–3%) and did not differ among experimental groups (Figure 7H). Analysis of MFI further supported these findings. Normalized MHC class I MFI was significantly increased only in *Pb*A‐infected mice (Figure 7I). Similarly, CXCL10 MFI was significantly elevated in *Pb*A‐ and coinfected mice, with the strongest increase observed following *Pb*A infection (Figure 7J), whereas TGFβ MFI remained unchanged across all groups (Figure 7K). Finally, co‐expression analysis demonstrated that the frequency of MHC class I^+^CXCL10^+^ microglia was significantly increased in *Pb*A‐ and coinfected mice compared with controls, consistent with an enhanced inflammatory phenotype (Figure 7L). In contrast, CD206^+^TGFβ^+^ microglia represented only approximately 1% of the total microglial population, and neither their frequency nor TGFβ MFI differed significantly between experimental groups (Figure 7M).

To determine whether similar changes occurred in infiltrating myeloid cells, brain monocytes were subsequently analyzed (Figure 8). Total CD11b^+^CD45^hi^ monocytes were significantly increased in all infected groups compared with uninfected controls, with the highest numbers observed in coinfected mice (Figure 8A). Subdivision according to Ly6C expression revealed an expansion of Ly6C^hi^ classical, Ly6C^int^ intermediate and Ly6C^low^ non‐classical monocytes, particularly during coinfection (Figure 8B–E). The absolute number of Ly6C^low^ monocytes was significantly increased following *Pb*A, *Tg*, and coinfection compared with controls, with the strongest increase observed in coinfected mice (Figure 8C). Ly6C^int^ monocytes were also significantly increased in all infected groups. This increase was modest following *Pb*A infection, more pronounced following *Tg* infection, and greatest during coinfection (Figure 8D). Similarly, Ly6C^hi^ monocytes accumulated significantly in the brains of all infected mice, with a moderate increase in the *Pb*A group and more marked increases in *Tg*‐infected and coinfected mice. The highest absolute numbers were observed during coinfection (Figure 8E). Together, these results indicate that infection promotes the recruitment of all Ly6C‐defined monocyte populations, with coinfection producing the most pronounced overall accumulation.

**FIGURE 8:**
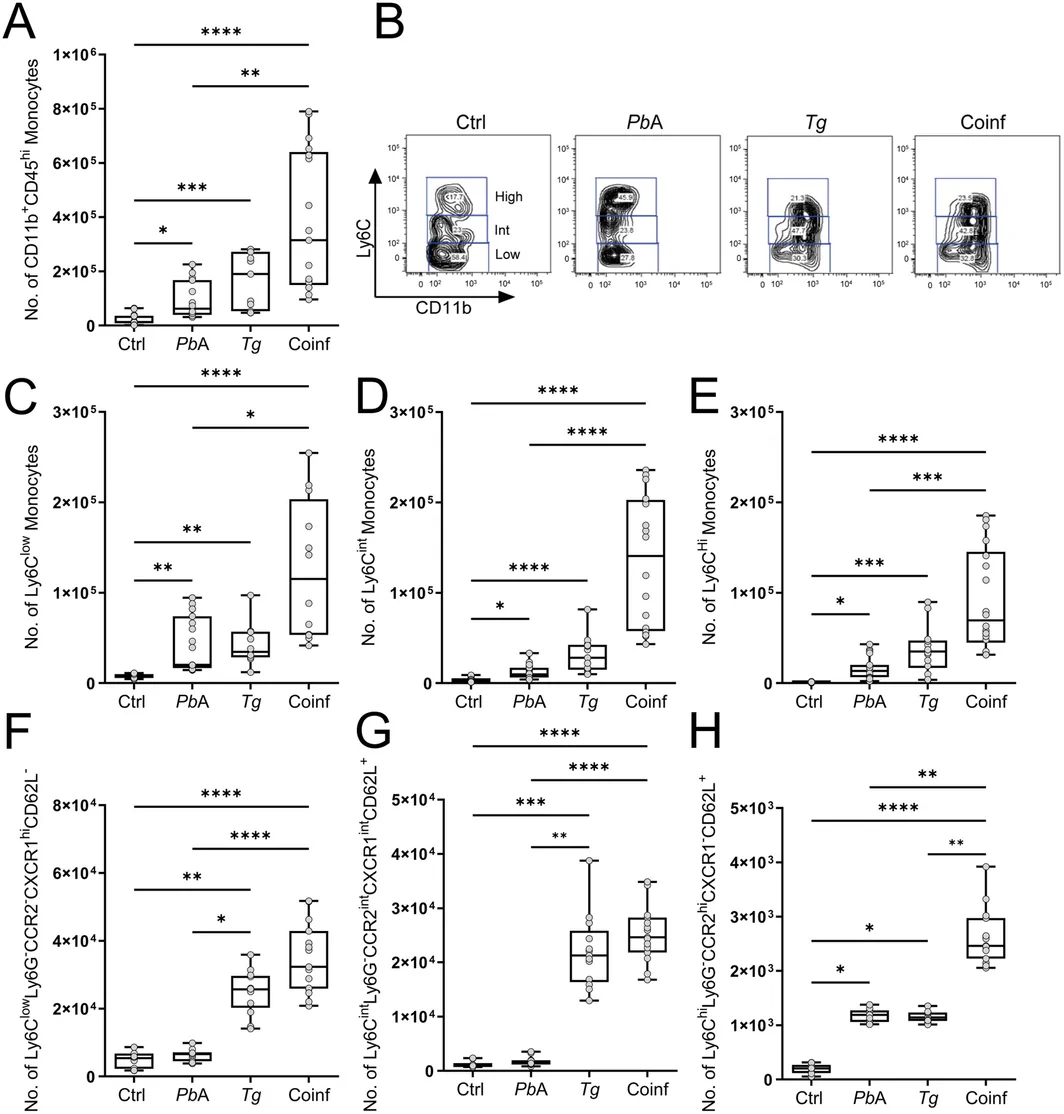
Enhanced recruitment of monocyte subsets in brain during the *Tg*‐*Pb*A coinfection. (A) Quantification of CD11b^+^CD45^hi^ monocytes in the brain. (B) Gating strategy for Ly6C subsets. (C–E) Quantification of Ly6^hi^, Ly6C^int^, and Ly6C^low^ monocytes. (F–H) Characterization of patrolling (Ly6C^low^Ly6G^−^CCR2^−^CX3CR1^hi^CD62L^−^), intermediate (Ly6C^int^Ly6G^−^CCR2^int^CX3CR1^int^CD62L^+^) and inflammatory (Ly6C^hi^Ly6G^−^CCR2^hi^CX3CR1^−^CD62L^+^) monocyte subsets. Coinfection is associated with increased recruitment of multiple monocyte subsets compared to single infections. Data are presented as box‐and‐whisker plots showing the median (center line), the 25th–75th percentiles (box), and the minimum and maximum values (whiskers), with individual data points displayed (*n* = 8–20, three independent experiments). Statistical analyses were performed using the Kruskal–Wallis test followed by Dunn's multiple‐comparison test. *p < 0.05; **p < 0.01; ***p < 0.001; ****p < 0.0001.

To further characterize these populations, monocytes were classified as non‐classical patrolling (Ly6C^low^Ly6G^−^CCR2^−^CX3CR1^hi^CD62L^−^), intermediate (Ly6C^int^Ly6G^−^CCR2^int^CX3CR1^int^CD62L^+^), and inflammatory (Ly6C^hi^Ly6G^−^CCR2^hi^CX3CR1^−^CD62L^+^) subsets. Coinfected mice exhibited substantial recruitment of all three monocyte subsets into the brain (Figure 8F–H). Patrolling Ly6C^low^ monocytes were virtually absent in mice with ECM but were significantly increased in *Tg*‐infected and coinfected mice (Figure 8F), indicating that their recruitment is primarily associated with chronic *T. gondii* infection and is not further enhanced by secondary *Pb*A infection. Similarly, Ly6C^int^ intermediate monocytes accumulated in the brains of *Tg*‐infected and coinfected mice, with no significant difference between these two groups, suggesting that their recruitment is also predominantly driven by chronic *Tg* infection (Figure 8G). In contrast, Ly6C^hi^ inflammatory monocytes were significantly increased in both ECM and chronic *Tg* infection and were further expanded during coinfection (Figure 8H), indicating that *Pb*A and *Tg* together promote enhanced recruitment of inflammatory monocytes into the brain. Overall, these findings demonstrate that *Tg*–*Pb*A coinfection promotes both robust microglial activation and selective recruitment of inflammatory monocyte subsets, contributing to the establishment of a distinct neuroinflammatory environment.

## 4 Discussion

Our comprehensive investigation of the coinfection dynamics between *Toxoplasma gondii* (*Tg*) and *Plasmodium berghei* ANKA (*Pb*A) provides critical mechanistic insights into the neuroprotective phenomena observed in experimental cerebral malaria (ECM). Employing the B6 experimental model, we demonstrate that the pathophysiological mechanisms underlying chronic *Tg* infection—characterized by the establishment of dormant bradyzoite‐containing tissue cysts within neural parenchyma—interact functionally with the neuroinflammatory cascades implicated in ECM pathogenesis (Blanchard et al. 2015; Nasuhidehnavi and Yap 2021; Suzuki 2020). These findings highlight the central role of glial cell plasticity in shaping neuroimmune responses during polymicrobial challenges affecting the CNS.

Our findings support the emerging concept that chronic *T. gondii* infection establishes a disease‐tolerant neuroimmune environment that can modulate the outcome of subsequent CNS infections (Osborne et al. 2014; Abu‐Raddad et al. 2006). In this context, we demonstrate that chronic cerebral *T*g infection profoundly reshapes the neuroinflammatory milieu during ECM. This study provides insight into the complex interactions between these two parasites and how they reshape host neuroimmune responses, ultimately influencing pathological trajectories and survival outcomes in polymicrobial infections. The establishment of chronic cerebral *T. gondii* infection was required for protection against ECM, as prevention of cerebral parasite persistence by sulfadiazine abolished the protective phenotype. Although peripheral parasitemia, cerebral *PbA* burden, and sequestration of infected erythrocytes remained comparable between *Pb*A‐infected and coinfected mice, these findings suggest that, within our experimental conditions, local neuroimmune regulation within the CNS contributes to protection in the absence of detectable changes in parasite burden or vascular sequestration. However, because sulfadiazine treatment prevents both cerebral parasite persistence and ongoing parasitism, these experiments do not formally distinguish local from systemic effects. Importantly, preservation of BBB integrity and reduction of cerebral edema in coinfected mice represent key physiological correlates of this regulated neuroinflammatory state. These physiological changes provide a functional correlate of the regulated neuroimmune environment established during coinfection. Because *Pb*A‐infected mice were analyzed at the onset of ECM whereas coinfected mice remained clinically protected, the observed molecular and cellular differences should be interpreted as being associated with the protected phenotype rather than demonstrating direct causal mechanisms. Future studies using clinical score‐matched animals or longitudinal analyses will be required to distinguish early protective events from secondary changes associated with disease progression. These observations are consistent with an important contribution of local neuroimmune regulation to the protective phenotype, although they do not exclude additional contributions from systemic immune responses. Taken together, these observations support the hypothesis that protection from ECM is associated with modulation of the host neuroimmune response rather than detectable alterations in parasite burden or vascular sequestration.

Building upon the prerequisite spatial requirements, we identified molecular mechanisms pathways associated with the neuroprotective phenotype observed during coinfection. Surviving coinfected mice exhibited distinct neuroimmune transcriptional profiles in which pro‐inflammatory mediators remained expressed while regulatory pathways involving TGF‐β and IL‐33/ST2 were preferentially enhanced. Importantly, IL‐10 expression was increased in all infected groups, indicating that this cytokine is not specific to chronic *T. gondii* infection. Rather than reflecting a purely anti‐inflammatory response, the transcriptional profile associated with chronic *T. gondii* infection combined inflammatory and regulatory components, consistent with previous studies showing that persistent parasite control requires sustained Th1 immunity, including TNF‐α and IFN‐γ production (Blanchard et al. 2015; Wilson and Hunter 2004; Nasuhidehnavi and Yap 2021). This balanced neuroimmune state may limit excessive neuroinflammation while preserving effective antimicrobial defense. CXCL‐10, a chemokine strongly induced during *Pb*A infection and instrumental in orchestrating pathogenic CD8^+^ T cell recruitment to the brain during ECM, remained expressed during coinfection but at substantially lower levels than in *Pb*A‐infected mice (Dalko et al. 2016, 2015; Leleu et al. 2022). This moderated expression may contribute to limiting pathogenic neuroinflammation while preserving immune surveillance. In parallel, the increased expression of TGF‐β, a pleiotropic cytokine involved in tissue repair and the resolution of inflammation, further supports the establishment of a regulated neuroimmune environment (Cekanaviciute et al. 2014). Collectively, these findings support the concept that chronic *T. gondii* infection does not suppress neuroinflammation but rather reshapes it toward a regulated neuroimmune equilibrium combining inflammatory and regulatory pathways. Such a balanced response may contribute to limiting ECM‐associated pathology while maintaining effective antiparasitic immunity. These observations are consistent with previous studies highlighting the complementary roles of IL‐10 and TGF‐β in controlling neuroinflammatory responses while preserving neural tissue integrity (Cekanaviciute et al. 2014; Keswani and Bhattacharyya 2014; Deininger et al. 2000; Kwon and Koh 2020).

Astrocytes are highly plastic glial cells that dynamically adapt their phenotype in response to environmental cues (Patani et al. 2023). Rather than supporting a strict A1/A2 dichotomy, our data identify the emergence during coinfection of reactive astrocytes displaying an intermediate inflammatory phenotype characterized by the concomitant expression of inflammatory (CXCL‐10, MHC‐I) and regulatory (TGF‐β) markers. This mixed activation state is consistent with a regulated astrocyte response capable of limiting excessive neuroinflammation while preserving local antimicrobial functions. This interpretation is supported by the multidimensional flow cytometry analyses showing the coexistence of astrocyte populations co‐expressing inflammatory and regulatory markers rather than mutually exclusive activation states.

In our study, we observed an increase in the number of astrocytes and microglia in the brains of mice coinfected with *Pb*A and *Tg* compared to uninfected controls. Interestingly, the abundance of these glial cells in coinfected mice was lower than in mice mono‐infected with *Tg*, correlating with the tendency of a reduced number of *Tg* cysts observed in the brains of coinfected mice. This suggests that *Pb*A coinfection influences *Tg* replication, potentially through modulation of the local immune environment. Notably, the reduction in cerebral *Tg* cyst burden observed at 27 dpi in coinfected mice should not be interpreted as complete immune‐mediated parasite clearance, as residual cyst structures and parasite transcripts remained detectable within the brain parenchyma. Instead, this finding is more consistent with partial limitation of cyst expansion or maintenance during coinfection. Several non‐mutually exclusive mechanisms may account for this phenomenon. First, enhanced inflammatory signals induced by *Pb*A infection may transiently increase immune pressure on *Tg* cysts, restricting parasite persistence without eliminating the infection. Second, alterations in glial activation states and local cytokine production may modify the cellular niches required for long‐term cyst stability. Third, systemic metabolic or immunological competition between *Pb*A and *Tg* may indirectly influence parasite survival within the CNS. Collectively, these mechanisms suggest that the reduced cyst burden reflects a dynamic host–parasite equilibrium rather than eradication of the parasite.

Critically, *Tg*‐*Pb*A coinfected mice displayed astrocytes exhibiting an intermediate inflammatory phenotype, characterized by the co‐expression of inflammatory (CXCL‐10, MHC‐I) and regulatory (TGF‐β) markers. Furthermore, reduced activation of glial cells in coinfected brains, characterized by an intermediate M1/M2 microglial activation profile, suggests the establishment of a regulated neuroinflammatory state. This intermediate glial phenotype represents a hallmark of functional glial plasticity, enabling the CNS to limit excessive inflammation while preserving antimicrobial defense mechanisms. These findings suggest that moderated glial response in coinfected mice may simultaneously limit *Tg* replication and prevent the neuropathological consequences of *Pb*A infection. The increased recruitment of diverse monocyte subsets during coinfection further supports the establishment of a regulated inflammatory environment, potentially contributing to both parasite control and limitation of tissue damage, in coordination with local glial responses. These observations suggest that coordinated astrocyte and myeloid cell responses contribute to the establishment of a regulated neuroimmune environment during coinfection.

Building upon the astrocyte remodeling observed during coinfection, we identified the IL‐33/ST2 axis as a neuroimmune pathway associated with the regulated inflammatory environment established within the brain. Reactive astrocytes represented one of the major IL‐33‐expressing cell populations identified in the brains of *Tg*‐infected and coinfected mice and were associated with increased recruitment of ILC2s within the cerebral parenchyma. Consistent with previous studies, IL‐33 functions as an important mediator of neuroimmune interactions, acting as a damage‐associated molecular pattern (DAMP) and promoting ILC2 recruitment and activation through its receptor ST2 (Still et al. 2020; Zhou et al. 2014). The IL‐33/ST2 signaling axis has also been shown to promote the expansion of IFN‐γ^+^ T lymphocytes, iNOS^+^ monocytes, and ILC2s, thereby contributing to the control of *Tg* infection and host defense within the CNS (Still et al. 2020). Recruited ILC2s subsequently produce IL‐13 and IL‐5, cytokines that support tissue protection and the modulation of neuroinflammation (Yeung et al. 2021). Importantly, in our study, the marked reduction in cerebral ILC2 recruitment observed in IL‐33^−/−^ mice was associated with only a partial loss of protection against ECM, indicating that the IL‐33/ST2 pathway contributes to, but is not solely responsible for, the establishment of disease tolerance during coinfection. Together, these findings identify the IL‐33/ST2 axis as an important interface between reactive astrocytes and infiltrating immune cells. Rather than representing a fully autonomous regulatory circuit, this pathway is more likely to operate within a coordinated neuroimmune network involving astrocytes, myeloid cells, and lymphoid populations that collectively contribute to maintaining controlled neuroinflammation and preserving BBB integrity during coinfection.

Our observations align with extensive clinical evidence demonstrating that coinfections substantially modulate immune responses and disease outcomes, particularly in endemic regions where multiple pathogens exhibit co‐transmission patterns (Galani et al. 2021; Hussein et al. 2022; Luz et al. 2018; Cox 2001; Rattanapunya et al. 2015). The mechanistic insights from our model provide a framework for understanding these clinical observations and suggest that apparent disease resistance in certain populations may reflect underlying coinfection dynamics rather than genetic resistance alone. The *Tg*‐*Pb*A coinfection model provides a valuable experimental paradigm for investigating CNS disorders characterized by neuroinflammation and BBB dysfunction, with potential implications for understanding diseases such as multiple sclerosis and Alzheimer's disease.

One limitation of this study is that our conclusions rely primarily on a murine model of coinfection, which may not fully capture the complexity of human neuroparasitic interactions in endemic settings. In addition, although our data identify reactive astrocytes as one of the major IL‐33‐expressing cell populations associated with the regulated neuroimmune environment and ILC2 recruitment during coinfection, the present study does not fully establish the causal contribution of the IL‐33/ST2 pathway to protection against ECM. Further studies using astrocyte‐specific genetic models, ST2 blockade, ILC2 depletion, cytokine neutralization, or complementary gain‐of‐function approaches will be required to define the precise contribution of the IL‐33/ST2 axis and downstream immune pathways to disease tolerance during coinfection. In particular, astrocyte‐specific IL‐33 conditional knockout models will be required to distinguish the contribution of astrocyte‐derived IL‐33 from that of other CNS IL‐33‐producing cell populations, including endothelial cells and oligodendrocytes.

The neuroimmune pathway identified in this study—from parasitic co‐localization through astrocyte remodeling to IL‐33/ST2/ILC2 circuit activation—suggests several potential therapeutic targets. The IL‐33/ST2 pathway represents a promising therapeutic target for diseases characterized by severe neuroinflammation, including CM. Therapeutic modulation of this pathway may provide novel strategies for disease progression control in CNS‐related disorders (Glineur et al. 2022).

In conclusion, our findings support a model in which chronic *Tg* infection establishes a preconditioned neuroimmune environment that reshapes the neuroimmune landscape through astrocyte remodeling and is associated with IL‐33/ST2 signaling. This coordinated neuroimmune response preserves BBB integrity, limits pathogenic neuroinflammation, and maintains controlled parasite persistence, thereby contributing to protection against fatal ECM during coinfection.

## Author Contributions

**S.P., S.M.:** study concept and design. **I.L., T.K., F.H., C.P., M.A.:** performed the experiments. **I.L.:** statistical analysis. **I.L., T.K., F.H., S.M.:** contributed to reagents/materials/analysis tools. **I.L., T.K.:** acquisition and interpretation of data. **I.L., T.K., C.G., S.M., S.P.:** contributed to writing of the manuscript.

## Funding

This work was supported by the Alliance Française contre les Maladies Parasitaires (LabEx PARAFRAP) (grant no. ANR‐11‐LABX‐0024).

## Conflicts of Interest

The authors declare no conflicts of interest.

## Supporting information


**Table S1:** List of primers used for RT‐qPCR analysis.

**Table S2:** List of antibodies used in flow cytometry.

**Figure S1:** Gating strategy for identification of ILCs subsets by flow cytometry. ILC1 were labeled by Lineage^−^RORγt^+^T‐bet^+^; ILC2 by Lineage^−^CD127^+^RORγt^−^GATA3^+^; and ILC3 by Lineage^−^CD127^+^RORγt^+^GATA3^−^ IL‐22^+^/NKp46^+^Tbet^+^. The final gates show intracellular detection of IL‐5 and IL‐13 within the gated brain ILC2 population.

## Data Availability

Data will be made available on request.
